# Stepwise-Nanocavity-Assisted Transmissive Color Filter Array Microprints

**DOI:** 10.1155/2018/8109054

**Published:** 2018-09-02

**Authors:** Yasi Wang, Mengjie Zheng, Qifeng Ruan, Yanming Zhou, Yiqin Chen, Peng Dai, Zhengmei Yang, Zihao Lin, Yuxiang Long, Ying Li, Na Liu, Cheng-Wei Qiu, Joel K. W. Yang, Huigao Duan

**Affiliations:** ^1^State Key Laboratory of Advanced Design and Manufacturing for Vehicle Body, College of Mechanical and Vehicle Engineering, Hunan University, Changsha 410082, China; ^2^School of Physics and Electronics, Hunan University, Changsha 410082, China; ^3^SZU-NUS Collaborative Innovation Center for Optoelectronic Science & Technology, International Collaborative Laboratory of 2D Materials for Optoelectronics Science and Technology of Ministry of Education, College of Optoelectronic Engineering, Shenzhen University, Shenzhen 518060, China; ^4^Department of Electrical and Computer Engineering, National University of Singapore, 4 Engineering Drive 3, Singapore 117583, Singapore; ^5^Engineering Product Development Pillar, Singapore University of Technology and Design, 8 Somapah Road, Singapore 487372, Singapore; ^6^Kirchhoff Institute for Physics, University of Heidelberg, Im Neuenheimer Feld 227, 69120 Heidelberg, Germany

## Abstract

Visible-light color filters using patterned nanostructures have attracted much interest due to their various advantages such as compactness, enhanced stability, and environmental friendliness compared with traditional pigment or dye-based optical filters. While most existing studies are based on planar nanostructures with lateral variation in size, shape, and arrangement, the vertical dimension of structures is a long-ignored degree of freedom for the structural colors. Herein, we demonstrate a synthetic platform for transmissive color filter array by coordinated manipulations between height-varying nanocavities and their lateral filling fractions. The thickness variation of those nanocavities has been fully deployed as an alternative degree of freedom, yielding vivid colors with wide gamut and excellent saturation. Experimental results show that the color-rendering capability of the pixelated nanocavities can be still retained as pixels are miniaturized to 500 nm. Crosstalk between closely spaced pixels of a Bayer color filter arrangement was calculated, showing minimal crosstalk for 1 *µ*m^2^ square subpixels. Our work provides an approach to designing and fabricating ultracompact color filter arrays for various potential applications including stained-glass microprints, microspectrometers, and high-resolution image sensing systems.

## 1. Introduction

Transmissive color filters, with their ability to selectively transmit a portion of the incident light, are key elements in various products from stained glass to optoelectronic systems such as digital display spectrometers, complementary metal oxide semiconductor (CMOS) image sensors, and liquid crystal display devices [1–6]. In the past decade, transmissive color filters based on plasmonic nanostructures have provided a promising alternative to conventional pigment-based printing due to their advantages in compactness, monolithic integration, enhanced stability, and environmental friendliness [2, 7]. With the unique optical properties of subwavelength plasmonic structures [8, 9], transmissive color filters with high transmission efficiency and color tunability can be achieved by just varying the size, shape, and arrangement of the metallic nanostructures [2, 10–14]. The potential for applications in consumable optoelectronic devices stimulated efforts to improve color saturation and transmission efficiency,* e.g.,* via optimizing the materials and structure design of the filters [15–18]. However, due to the intrinsic metal losses of the plasmonic nanostructures, color purity and transmission efficiency are difficult to be achieved simultaneously to satisfy the performance requirements for high-end optoelectronic device applications [19–22]. Besides, from the structural design perspective, the colors of existing demonstrated color filters are mostly achieved by tuning the planar parameters of nanostructures, while the vertical dimension of the structures has not been well explored as a degree of freedom to tune the structural colors. The challenges associated with precise spatial variation and control of the height of nanostructures account for the difficulty in realizing color elements based on pixel height variation [23].

It is well known that wide gamut of colors can also be obtained by interference effects in multilayer thin films [7, 24–26]. By carefully choosing the materials and thicknesses of the thin films [27], different transmissive color filters with desired wavelength range, linewidth, and transmission intensity can be achieved. These color filters are widely used in existing optical systems such as phototransistors, modulators, and amplifier [28–30]. Among various designs, metal-dielectric-metal Fabry-Pérot (MDMFP) cavity is the simplest configuration considering the ease of design and fabrication [25, 31, 32]. Compared to the color filters achieved by planar plasmonic nanostructures, FP cavity-based color filters are tuned by varying the thicknesses of the dielectric layer and thus provide a degree of freedom to tune the colors using the vertical dimension of structures. FP cavity-based color filters also promise better transmission efficiency, wider color gamut, and narrower spectral linewidth compared to plasmonic color filters, which is preferable for applications [28, 33]. For example, compared with common plasmonic color filters, Ag/SiO_2_/Ag triple layer FP cavities have shown highly tunable colors with a linewidth of ~25 nm and transmission efficiency up to 50% [25, 32]. Despite these advantages, forming pixelated cavities for use in monolithic color filter arrays (CFAs) is challenging due to the limitations of thin-film deposition processes. Even if multiple film deposition or etching rounds are adopted to achieve monolithic integration of different color filters [34–36], the pixel size of the CFAs is commonly at the 100 *μ*m scale and the channel numbers are also limited, which cannot satisfy high-resolution integration requirement.

Recently, we demonstrated that the greyscale lithography process could be used to fabricate stepwise pixelated FP cavities for monolithic reflective CFAs [23]. The key idea in the proposed configuration is to realize different optical properties by varying the vertical dimension of nanostructures. By carefully controlling the exposure dose in an inorganic negative-tone hydrogen silsesquioxane (HSQ) electron-beam resist, stepwise SiO_x_ thin films could be directly defined to achieve FP cavities with varied dielectric thickness, which enabled the realization of reflective colors achieving wide gamut and contrast.

In this work, we evaluate the feasibility of using greyscale lithography to achieve pixelated transmissive CFAs via optimized design and fabrication processes. By replacing the SiO_2_ dielectric layer in a common Ag/SiO_2_/Ag sandwich FP cavity resonator using nanoscale-pixelated SiO_x_ obtained from exposed HSQ resist, high-resolution monolithic color filters with wide gamut and excellent saturation were obtained. Optimized material and structural designs including appropriate adhesion layer, top capping layer, and cavity filling density were chosen to satisfy the nanofabrication compatibility, increase the transmission efficiency, and improve the color gamut. Experimental results show that the transmissive CFAs based on FP cavities have a pixel resolution of 500 nm pitch. With this capability, arbitrary microprints with vivid colors can be fabricated. Finally, crosstalk between closely spaced pixels of a Bayer color filter arrangement was calculated, showing minimal crosstalk for 1 *μ*m^2^ square subpixels. Compared to plasmonic counterparts, the transmissive CFAs based on FP nanocavities in this work possess comparative merits of high-resolution and monolithic integration for compactness but have the advantages of enhanced transmission efficiency, narrower spectral linewidth, and better color tunability, which thus provide an alternative solution to design and fabricate high-performance transmissive CFAs for various applications including color displays, microspectrometers, and image sensors.

## 2. Results

**Figure 1:**
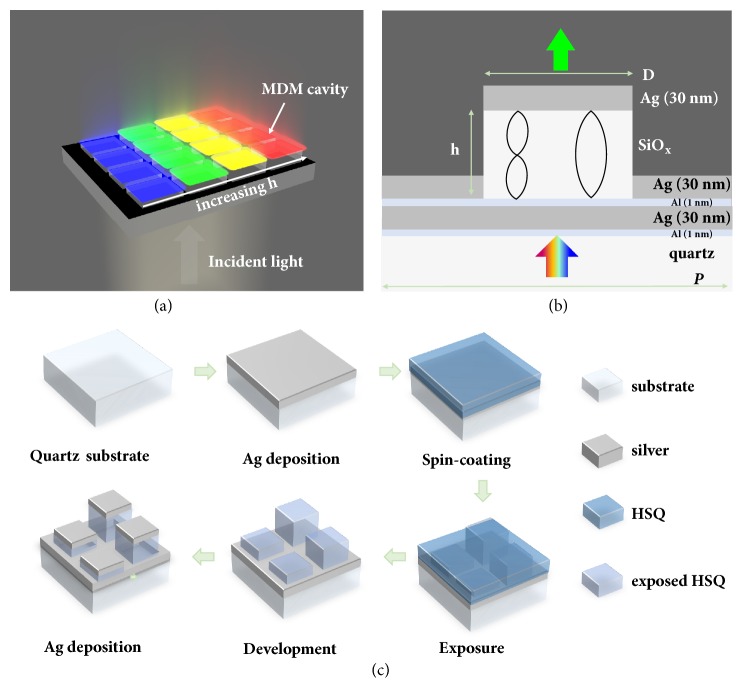
(a) Schematic of a transmissive color filter array based on stepwise FP cavities. (b) Schematic showing the cross-section profile of a unit of the FP cavity arrays, in which both the height (*h*) and the size (*D*) of SiO_x_ dielectric structures are variable parameters to tune the colors. (c) The fabrication process flow to obtain the color filter array.

The three-dimensional (3D) schematic diagram of the proposed transmission-type color filter is illustrated in Figure 1(a), and the corresponding cross-sectional profile is shown in Figure 1(b). The basic configuration of the FP cavity is a Ag/SiO_x_/Ag sandwich structure on the quartz substrate. By carefully designing the thickness of the sliver (Ag) layers and the dielectric SiO_x_ layer, different resonance wavelengths can be obtained due to the constructive interferences when varying the thickness* h* of the SiO_x_ layer, producing different transmission colors. Note that two ultrathin aluminum (Al) layers (1 nm) were used in the designed configuration to promote the adhesion of Ag to its underlying surface. Simulations indicate that the ultrathin aluminum adhesion layers only slightly decrease the transmission efficiency.

Figure 1(c) shows the step-by-step fabrication process for the CFAs. Starting from a quartz substrate, a 30-nm-thick Ag film was first deposited as the bottom metal layer. To obtain a wide range of colors, dielectric spacer layers with different thickness and filling densities were patterned by greyscale lithography using HSQ as a negative-tone resist. The utilization of HSQ resist is essential to this configuration because it transforms into transparent dielectric SiO_x_ layer after electron-beam exposure. By carefully controlling the exposure doses close to the cutoff dose of the contrast curve [37], we can control the thickness of patterned HSQ resist after the development, and these exposed HSQ patterns acted as the dielectric layer placed between two metal films. After depositing a 30-nm-thick Ag film top layer, an array of stepwise FP cavity resonators were formed, while the surface without HSQ patterns was covered by an opaque Ag film with a total thickness of 60 nm.

In addition to varying the thickness of the dielectric layer for color tuning, varying the filling factor (determined by the square size,* D*) of the HSQ nanopatterns with a fixed pixel pitch (*P*) can be used to fine-tune the transmission colors. According to the principle of constructive interference and equivalent refractive index [38], either adjusting the thickness or the filling factor of the defined HSQ nanostructures would generate various effective optical path differences, leading to distinct transmitted colors in the visible regime under white-light illumination.

**Figure 2:**
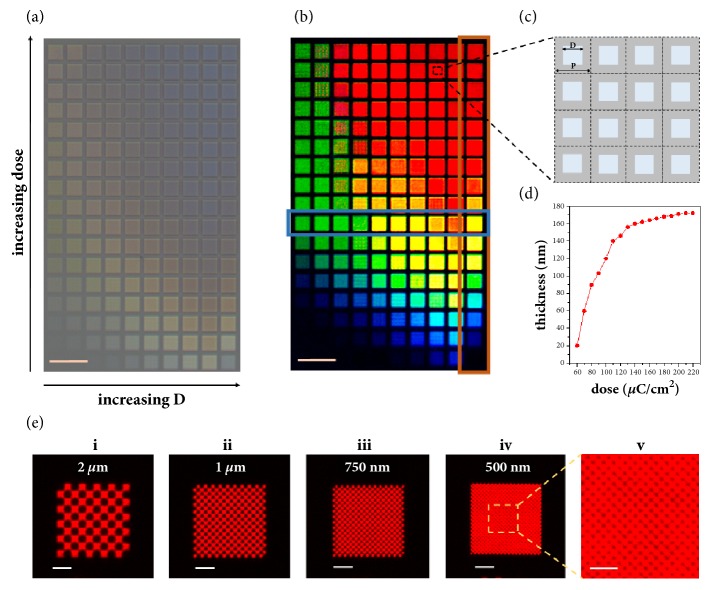
Transmission optical micrographs of patterned tiles with varied sizes* D *from left to right (along x axis; 331 nm ≤ D ≤ 500 nm) and thickness* h* (along y axis) of dielectric layer before (a) and after (b) top-layer Ag deposition. Each tile (20 *μ*m) consisted of 40 square-shaped pixels (*P *= 500 nm). (c) Schematics showing 16 pixels to provide intuitive concept of filling factor discussed below. (d) The thickness variation of spacer layer as a function of exposure dose corresponding to the red-color highlighted column in (b). (e) Checkerboard patterns with decreasing pixel size. Scale bars: 50 *μ*m (a, b), 5 *μ*m (e(i-iv)), and 2.5 *μ*m (e(v)).

As shown in Figure 2(a), no obvious colors were observed before depositing the top-layer Ag film. In contrast, vivid colors with high saturation and brightness were exhibited after the formation of the FP cavities by depositing the top-layer silver film, as shown by the color palette in Figure 2(b). In the palette, the size of each tile was 20 *μ*m. Each tile consisted of 40 × 40 square-shaped SiO_x_ nanopillars with a pitch of* P* = 500 nm as the subpixels. The filling factors of the subpixels were tuned by varying the size* D* of the nanopillars, as indicated by Figure 2(c). In the CFAs, the filling factor (FF,* i.e.,* the ratio of the total area of nanopillars in a tile) increased from 7/16 to 16/16 (*D* increased from 331 nm to 500 nm) along the x axis, and the height of the SiO_x_ nanopillars gradually increased along the y axis by increasing the exposure dose. Figure 2(d) gives the measured thickness (*h*) of the dielectric SiO_x_ layer as a function of the exposure dose, indicating that the height of the nanopillars varied from 20 nm to 170 nm when the exposure dose increased from 60 *μ*C/cm^2^ to 220 *μ*C/cm^2^ with an increment of 10 *μ*C/cm^2^. More details about the layout design are given in supplemental material [Sec supplementary-material-1]b.

To investigate the pixel resolution limit, Figure 2(e) shows a series of checkboard patterns with decreased pixel sizes from 2 *μ*m to 0.5 *μ*m and Figure 2(e)(v) shows that the detailed characteristic under higher magnification of a specified local area highlighted by the dotted box. Interestingly, even for a small pixel size of 0.5 *μ*m, the same red color was observed as that in the checkboard with 2 *μ*m pixel size, indicating that the color filtering function of this kind of FP cavities can well remain at the submicrometer scale for applications requiring high-resolution pixels. In addition, no obvious color variation in these tiles was observed when varying the numerical aperture of the objective lens, as shown in supplemental material [Sec supplementary-material-1].

**Figure 3:**
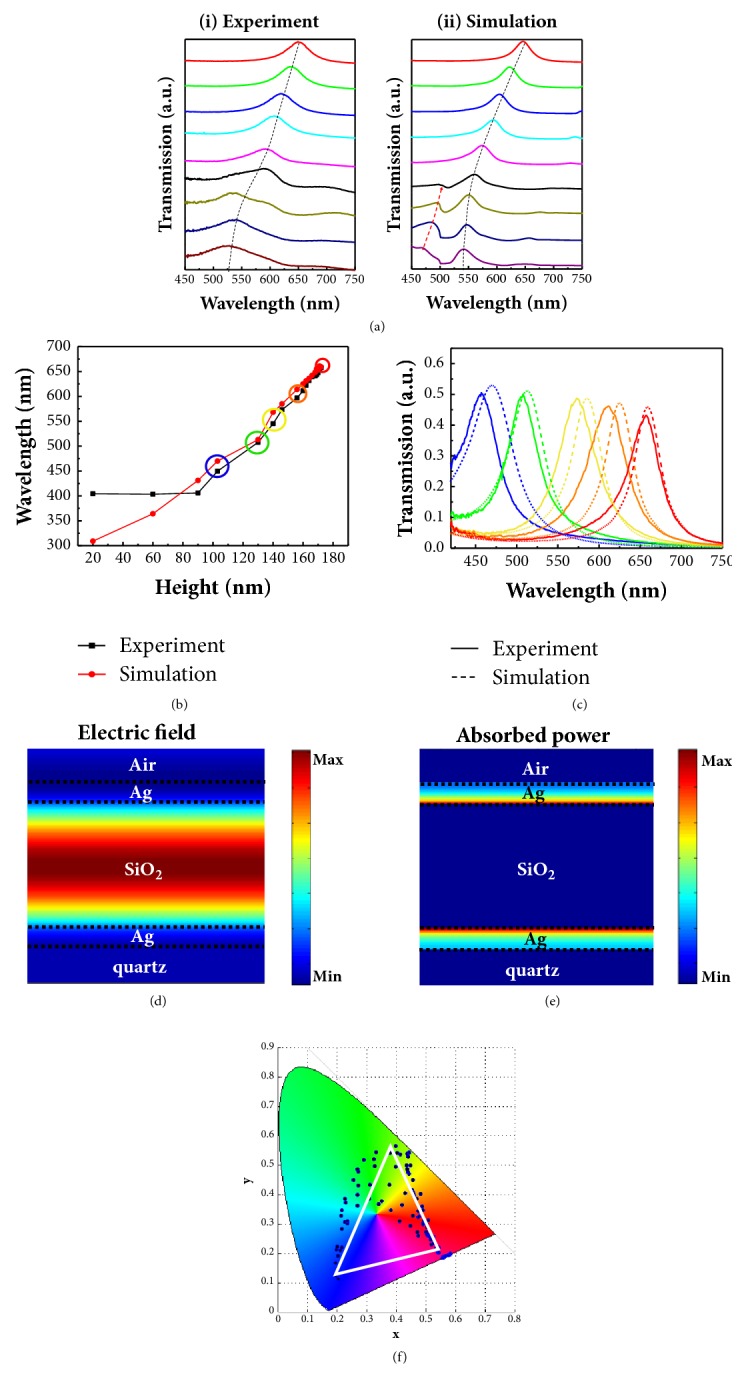
(a) Selected experimental (i) and simulated (ii) transmission spectra of the patterned tiles with varied size* D* of square-shaped SiO_x_ nanopillars ranging from 331 nm to 500 nm with a fixed height of 156 nm (the blue highlighted row in Figure 2(b)) under normal incidence. The dashed lines approximately indicate the shifting peaks when increasing the filling density* D*. (b) Experimental (black square scatter) and simulated (red circle scatter) transmission peak wavelengths as a function of the measured thickness of the dielectric layer with* D* = 500 nm (the red highlighted column in Figure 2(b)). (c) Five representative transmission spectra of the highlighted circles labelled in (b). (d, e) Cross-sectional electric field distribution (d) and absorbed-power profile (e) of the red spectrum (*h *= 172 nm) at the resonance wavelength* λ* = 650 nm. (f) CIE 1931 chromaticity coordinates of converted entire patterned colors showed in Figure 2(b).

To further understand the origin of the vivid colors spanning the whole visible region in Figure 2(b), the transmission spectra as a function of the varied parameters including the thickness and the filling factor of the dielectric layer were systematically analyzed.  Figure 3 shows both the experimental and simulated transmission spectra as well as the simulated near-field profiles of selected color pixels (highlighted by frames in Figure 2(b)). With a fixed dielectric thickness* h* of ~156 nm, Figure 3(a)(i) gives the transmission spectra of the tiles with different filling factor (indicated by the blue row in Figure 2(b)). Note that the spectra were normalized because we are solely interested in the peak position here. When the filling factor increases, the main transmission peak of the isolated pixel continuously redshifts along with the dashed line from ~ 525 nm to ~ 650 nm in the experimental measurements, which well agrees with the simulated transmission spectra depicted in Figure 3(a)(ii) and provides a degree of freedom to fine-tune the transmitted colors.

The dependence of the transmission colors on the filling factor is consistent with the understanding of effective refractive index. A higher filling fraction of dielectric pillars will have a larger effective refractive index and lead to increased optical path difference in the FP cavity, enabling the selected transmission of the light with a longer wavelength. Note that an additional peak (indicated by the red arrow) exists in the simulated spectra for the tiles with low filling factor and it gradually narrows and vanishes when increasing the filling factor. In-depth simulation analysis indicates that the appearance and vanishing of this peak are relevant to the lattice resonance of periodic structures such as the bottom periodic nanopits and top Ag nanocaps array, as discussed in supplemental material [Sec supplementary-material-1]. This peak was weak in the experimental spectra, which is presumably due to the imperfections of the fabricated dielectric nanopillars.

Stepwise cavity color filters containing full filling pixels (*i.e.,* FF = 16/16,* D* = 500 nm) are merely determined by the phenomena of constructive interference of multiple layer films.  Figure 3(b) shows a plot of the wavelengths at transmission peak as a function of SiO_x_ layer thickness embedded in FP cavity (indicated by a red box in Figure 2(b)). Due to the limited response and the poor signal to noise ratio of our spectrometer in the ultraviolet range, the optical response of cavity with 90 nm thickness interference layer, which is beyond 400 nm wavelength, was not well recorded in the plot. By increasing the thickness of SiO_x_ dielectric layer utilizing greyscale lithography, the peak of the transmission shifted to longer wavelength without sacrificing efficiency and a maximum transmittance up to 50% was achieved.  Figure 3(c) shows the measured (solid lines) and simulated (dash lines) transmission spectra of five selected tiles with different dielectric thickness (*h* = 103 nm, 130 nm, 146 nm, 160 nm, and 172 nm, respectively). In Figure 3(c), experimental spectra demonstrate qualitative agreement with the corresponding simulation results in terms of the overall shape and shifting trend. The full widths at half-maximum (FWHM) values for the measured transmission peaks are between 34 and 43 nm, implying high saturation of the generated colors. In all measured spectra, average transmission efficiency more than 40% was achieved. Both transmission efficiency and FWHM show much better performance compared to the plasmonic counterparts based on extraordinary transmission phenomenon. Furthermore, the transmission efficiency of the FP cavity could be further enhanced by coating a dielectric layer on the top silver thin film via better impedance matching, as shown and discussed in supplemental material [Sec supplementary-material-1]a and [Sec supplementary-material-1].

To further understand the transmission behavior of the FP cavity, the electric field distribution (Figure 3(d)) and absorption power profile (Figure 3(e)) of a common cavity with 172 nm dielectric thickness were plotted at the transmission peak wavelength of 650 nm. The electric field is highly confined at the SiO_x_ section embedded in the two Ag films, where a standing wave is formed and the optical power is gradually diminished from middle SiO_x_ layer to the Ag film of both sides. Under this cavity-resonance condition, destructive interference occurs for reflected waves, leading to minimum reflection, and constructive interference occurs for transmitted waves, leading to maximum transmission. However, due to the intrinsic loss in silver film, partial power was mainly absorbed by the silver at the two Ag/SiO_x_ interfaces, as indicated by Figure 3(e), resulting in a transmission efficiency of only ~50%. By changing the thickness of the dielectric SiO_x_ layer, the transmission resonance peak is highly tunable due to the varied phase delay of the light, which enables highly tunable colors. The chromaticity coordinates of the whole pixel colors of extended palette in Figure 2(b) have been captured spectrum from the spectrometer and plotted in the CIE 1931 color space diagram and are depicted in Figure 3(f), demonstrating the large degree of coloration capability of this configuration to cover the whole primary colors across the visible regime by manipulating the density and thickness of FP cavities.

**Figure 4:**
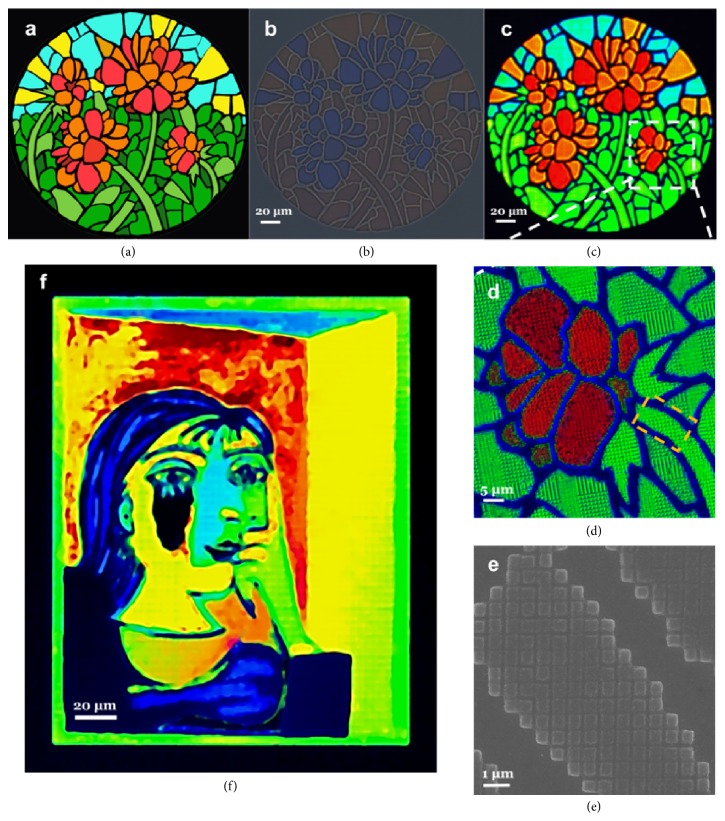
Reproducing vivid microprints using stepwise FP nanocavities. (a) The original water-colored painting* Summer Flowers*. (b, c) Transmission optical micrographs of patterned arrays before (b) and after (c) Ag capping layer deposition. (d) Detailed characteristic of the selected area by white dashed frame with higher magnification. (e) The corresponding SEM image of a typical area labelled by orange dashed frame in panel. (f) Full-color reproduction of a modified* Portrait of Dora Maar.*

To further highlight the wide gamut of this kind of transmission CFAs, we fabricated a water-color painting of* Summer Flowers* based on stepwise FP cavities, as shown in Figure 4(a). The original digital image was input into a MATLAB script that can search for the closest RGB color from the palette in Figure 2(b) to match the individual pixels for generating a predefined, square-like array of FP nanocavities with different thickness and spacing. Figure 4(b) shows an exposed pattern of 260 *μ*m × 260 *μ*m before metal deposition, in which subdivided tile comprises a quadratic unit cell with 500 nm periodicity, showing a rough outline with low saturation and brightness. After subsequent deposition of a uniform 30-nm Ag film top layer, a vivid painting with high fidelity was reproduced, as displayed in Figure 4(c). The detailed characteristic under higher magnification of a specified local area highlighted by the dotted box is presented in Figure 4(d), demonstrating the color consistency at higher magnification. Note that deep blue color was exhibited at the boundaries (which was supposed to be dark) of the color pieces due to the existence of a thin dielectric layer induced by the proximity effect in the EBL exposure process. In Figure 4(e), the corresponding enlarged SEM image of a steam leaf labelled by square dashed line in panel is shown. By varying the exposure dose factor to tune the thickness of FP cavities during the greyscale lithography process, we can obtain vivid paintings with subtle saturation variations (supplemental material [Sec supplementary-material-1]). To further verify the availability and expandability of this kind of transmissive CFAs for multicolor microprints, we accomplished a modified Picasso's painting of* Portrait of Dora Maar* (210 *μ*m × 170 *μ*m), as shown by Figure 4(f). Based on the reproduced paintings above, the proposed transmissive CFAs based on stepwise FP nanocavities show outstanding color distinction, tunability, and fidelity for printing vivid stained-glass microprints.

**Figure 5:**
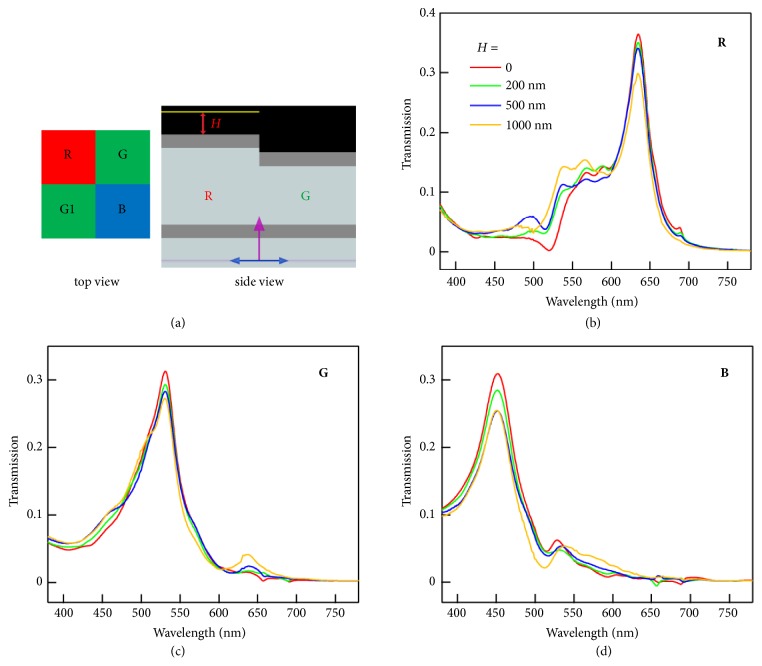
Crosstalk calculation in a FP cavity-based Bayer cell for high-resolution color image sensors. (a) Schematic of the FP cavity-based color filters in Bayer mosaic layout. The dielectric thicknesses of the Ag/SiO_x_/Ag FP cavity for the R, G (G1), and B pixels are 170 nm, 130 nm, and 100 nm, respectively. The size of each color pixel is 1 *μ*m × 1 *μ*m. Power monitors (yellow line) of the same size are placed right above each color pixel with varying distance* H*. (b-d) Simulated transmission spectra of the R (b), G (c), and B (d) pixels using monitors at different height* H*. The spectra are the average results obtained from normal incident plane waves of TE and TM polarization.

As one of the most attractive applications of transmissive CFAs is in high-resolution color image sensors, electromagnetic simulations were also carried out to investigate the color crosstalk behavior of FP nanocavities. When the size of a color subpixel decreases below 2 *μ*m, color crosstalk becomes one of the most serious issues of image sensors [39]. We consider the standard Bayer pattern, consisting of a unit cell with one red (R), one blue (B), and two green (G, G1) filters, which is also the most common design for color image sensors (Figure 5(a)) [18, 39, 40]. Two green filters are integrated in a Bayer unit owing to the higher sensitivity of the human visual system to the green portion of the light. Ag/SiO_x_/Ag FP cavities with dielectric thicknesses of 170 nm, 130 nm, and 100 nm are employed as R, G (G1), and B pixels, respectively. Both spectral and spatial crosstalk should be considered in the design of the color filters [39, 40]. Some amount of light in other color ranges passing through the imperfect color filters causes the spectral crosstalk. The spatial color crosstalk originates from the influence of adjacent pixels. For example, the red light transmitted through the red filter “leaks” to adjacent monitors above the green and blue filters, and vice versa. In our calculations, we set the size of the FP cavity at 1 *μ*m × 1 *μ*m, a relevant size for state-of-the-art commercial image sensors [39]. The calculations were performed with power monitors placed a distance* H* above the red (tallest) FP cavity. As shown in Figure 5, the FWHM values for the transmission peaks measured above the R, G, and B pixels are within 40 ~ 70 nm. The narrow peaks help reduce the spectral crosstalk of our FP cavity-based filters compared to the previously reported plasmonic filters [14, 16, 18, 39]. The spatial crosstalk is related to the distance between the filters and the photodiodes. When the monitor is further from the FP cavities, the intensity of the main transmission peak decreases and the transmission in other color ranges increases (Figures 5(b)–5(d)), resulting in increased spatial crosstalk.

To quantify the spatial crosstalk of each pixel separately, transmission spectra *T*_*i*_ of each pixel in revised Bayer cells were collected following procedures in a previous report [40]. A single Ag/SiO_x_/Ag FP filter in the revised Bayer cell is considered, while the other three pixels are blocked by replacing the SiO_x_ layers with Ag layers. When only the blue pixel is kept, the crosstalk of the light fluxes from the blue pixel to other pixels is defined as *C*_*Bi*_ and calculated as [40]

(1) $$\begin{array}{c} C_{Bi}=\frac{\int_{445}^{505}T_{i}d\lambda }{\int_{445}^{505}T_{B}d\lambda }\;i=G,G1,R \end{array}$$

Similarly, *C*_G*i*_, C_G1i_, and *C*_R*i*_ are calculated with the green and red spectral ranges defined as 515–575 nm and 595–655 nm, respectively. The calculated spatial optical crosstalk values with monitors placed at two typical heights are shown in Table 1. At* H* = 1 *μ*m, the FP filters in the Bayer cell exhibit significant spatial optical crosstalk, especially in the leakage between the red and green pixels. When the monitors are brought closer to the filters at* H* = 500 nm, the spatial color crosstalk is reduced. The calculated crosstalk values of our FP filters are comparable to those of plasmonic filters and pigmented filters with the same sizes [40]. Small distance is desirable for reducing spatial crosstalk when integrating the FP filters with photodiodes. Further optimization of our FP filters for practical high-resolution image sensors should also consider the illumination angle, the use of microlenses, and the accompanying signal processing [18, 39, 40]. Overall, our FP filters are promising alternatives to plasmonic filters and pigmented filters, since they exhibit small spectral crosstalk and moderate spatial crosstalk.

## 3. Conclusion

In conclusion, we have proposed and demonstrated a kind of monolithic transmissive CFAs based on stepwise Ag/SiO_x_/Ag FP nanocavities. By adjusting the filling density and thickness of the FP nanocavities with optimized structural design, vivid colors with wide gamut covering the entire visible-light range with excellent hue, saturation, and brightness were obtained. Furthermore, the proposed FP nanocavity shows the color-rendering capability down to 500 nm pixel size, which holds the potential for high-resolution colorization. Two vivid full-color microprints were demonstrated using this kind of transmissive CFAs. Bayer cells consisting of small FP filters were shown to possess competitive performance with small spectral and spatial crosstalk. Compared to the plasmonic nanostructures, the CFAs based on FP nanocavity have the advantages of enhanced color-rendering performance and can be extended for large-area production using greyscale photolithography. Thus, our work provides an alternative solution to design and fabricate ultracompact color filter arrays which may find broad applications for microprints, microspectrometers, and high-resolution image sensing systems.

## 4. Method

### 4.1. Electron-Beam Lithography

A 170-nm-thick HSQ resist (Fox-16, Dow Corning, diluted by MIBK) is spin-coated on the evaporated silver thin film atop quartz substrate. Subsequently, stepwise nanopatterns with different thickness and size were defined by Raith-150^two^ electron-beam lithography system at an accelerating voltage of 15 kV and beam current of 184 pA. Different doses were used to achieve the greyscale nanopatterns. After the exposure, the sample was developed by tetramethylammonium hydroxide (TMAH) at 20°C for 4 min and then rinsed with deionized (DI) water for 1 min and finally blow-dried by nitrogen (N_2_) stream.

### 4.2. Metal Deposition

Metal films were deposited using an electron-beam (E-beam) evaporator (Kurt J. Lesker, Lab-line). To obtain smooth thin films, the pressure of the evaporation chamber was prepumped down to 5 × 10^−7^ Torr. Meanwhile, the working pressure during evaporation was less than the value of 6 × 10^−6^ Torr. The Al adhesion layer (1 nm thick) and bottom Ag film (30 nm) were deposited on the top of a quartz substrate at a rate of 0.10 Å/sec and 0.45 Å/sec, respectively. After greyscale electron-beam patterning, 30-nm-thick top-layer Ag film was deposited onto the patterned nanosquares with a rate of 0.35 Å/sec.

### 4.3. Morphology Characterizations

Atom force microscopy (AFM, Bruker, MultiMode 8) working in tapping mode was utilized to evaluate the thickness and surface roughness of the fabricated color filter. A field-emission scanning electron microscope (Sigma HD, Carl-Zeiss) was used to characterize the surface morphology of the nanopatterns with an accelerating voltage of 10 kV and a working distance of 7 mm.

### 4.4. Optical Characterization

The transmittance spectra under normal incidence were acquired by Olympus microscope (BX-51) equipped with a spectrometer through a 100 × objective (MPlan-FLN, NA = 0.9). An optical microscope (Carl-Zeiss AXIO-10) with ×5 (0.13 NA), ×10 (0.25 NA), ×20 (0.4 NA), ×50 (0.75 NA), and ×100 (0.85 NA) objective lens is used to obtain the transmission optical micrographs with different magnifications.

### 4.5. Numerical Simulations

Three-dimensional finite-difference time-domain (FDTD) method (Lumerical Solutions Inc., Version 8.15) was used to calculate the optical responses and near-field distribution profiles of the proposed structures. The dispersion models of SiO_2_ and Ag were based on the data from Palik [41] in the material library of software. A unit cell of 0.5 *μ*m was used for calculating the transmission of single FP filters, while four 1 *μ*m × 1 *μ*m filters were included in the Bayer cell. Periodic boundary conditions along x- and y-axes and perfectly matched layers (PML) along the propagation of electromagnetic waves (z-axis) were used. The source was a broadband plane wave. A refinement mesh with 5-nm-size in in-plane direction and 2-nm-size in z direction was set to achieve the optimal results. The cross-section electric field and the absorbed-power distribution were obtained by two-dimensional Y-normal type frequency-domain field profile monitors. The different HSQ thicknesses were referenced to the AFM measurement. In addition, angular-dependence spectra in supplemental material were calculated by a BFAST type plane wave and Bloch boundary conditions along x- and y-axes.

## Figures and Tables

**Table 1:** Calculated spatial color crosstalk of FP filters in Bayer cell.

	*H* = 500 nm				*H* = 1 *μ*m			
	*C*_B*i*_ (%)	*C*_G*i*_ (%)	*C*_G1*i*_ (%)	*C*_R*i*_ (%)	*C*_B*i*_(%)	*C*_G*i*_ (%)	*C*_G1*i*_ (%)	*C*_R*i*_ (%)
B	/	12.5	12.5	4.6	/	15.2	15.2	8.5
G	9.2	/	1.5	12.3	14.0	/	4.6	21.5
G1	9.2	1.5	/	12.3	14.0	4.6	/	21.5
R	1.0	11.4	11.4	/	1.9	23.7	23.7	/

## Supplementary Materials

Supplementary Materials Figure S1: (a) optical micrographs of the obtained color palette with SiO2 coating. Scale bars: 50 *µ*m. (b) Schematic diagram of definition rule for the filling density D size with each pixel size of 500 nm. Figure S2: (a) optical micrographs of the obtained color palette under different numerical apertures. The specific numerical apertures are (i) 0.25 NA; (ii) 0.4 NA; (iii) 0.75 NA; and (iv) 0.85 NA. Scale bars: 25 *μ*m (i-iv). (b) Simulated transmission of the color filter for TE with various incidence angles (12°-30°). Figure S3: calculated transmission spectra of FP cavities (corresponding to the samples shown in Figure 3(a)) by adjusting the filling density of asymmetric FP cavities, but without the top Ag coating at normal incidence. Figure S4: (a) simulation and (b) experimental transmission spectra of asymmetric FP cavities with varying h and the condition of D = 500 nm (the red highlighted column in Figure 2(b)), and the configuration with top SiO2 coating layer (dots) and without top SiO2 coating (solid lines) are shown. Figure S5: the pixel size of the printing is uniform D = 500 nm, (a) the exposure dose is varied from 42 *μ*C/cm2 to 154 *μ*C/cm2 at a step dose of 10 *μ*C/cm2 and (b) the printing by varying from 48 *μ*C/cm2 to 176 *μ*C/cm2, and (c) the exposure dose is obtained from 54 *μ*C/cm2 to 198 *μ*C/cm2.
