# Supplementary material for: Stepwise-Nanocavity-Assisted Transmissive Color Filter Array Microprints
Source: Research (Wash D C). 2018 Sep 2;2018:8109054. doi: 10.1155/2018/8109054 (PMC6750064; doi:10.1155/2018/8109054)
Supplement: Supplementary Materials — Figure S1: (a) optical micrographs of the obtained color palette with SiO2 coating. Scale bars: 50 µm. (b) Schematic diagram of definition rule for the filling density D size with each pixel size of 500 nm. Figure S2: (a) optical micrographs of the obtained color palette under different numerical apertures. The specific numerical apertures are (i) 0.25 NA; (ii) 0.4 NA; (iii) 0.75 NA; and (iv) 0.85 NA. Scale bars: 25 μm (i-iv). (b) Simulated transmission of the color filter for TE with various incidence angles (12°-30°). Figure S3: calculated transmission spectra of FP cavities (corresponding to the samples shown in Figure 3(a)) by adjusting the filling density of asymmetric FP cavities, but without the top Ag coating at normal incidence. Figure S4: (a) simulation and (b) experimental transmission spectra of asymmetric FP cavities with varying h and the condition of D = 500 nm (the red highlighted column in Figure 2(b)), and the configuration with top SiO2 coating layer (dots) and without top SiO2 coating (solid lines) are shown. Figure S5: the pixel size of the printing is uniform D = 500 nm, (a) the exposure dose is varied from 42 μC/cm2 to 154 μC/cm2 at a step dose of 10 μC/cm2 and (b) the printing by varying from 48 μC/cm2 to 176 μC/cm2, and (c) the exposure dose is obtained from 54 μC/cm2 to 198 μC/cm2. [file 8109054.f1.docx]

Stepwise-nanocavity-assisted Transmissive Color Filter Array Microprints

Yasi Wang^1,#^, Mengjie Zheng^2,#^, Qifeng Ruan^3,4,5,#^, Yanming Zhou^2^, Yiqin Chen^1^, Peng Dai^2^, Zhengmei Yang^2^, Ying Li^3^, Na Liu^6^, Cheng-Wei Qiu^3,4^, Joel K. W. Yang^5,^*, Huigao Duan^1,^*

^1^ State Key Laboratory of Advanced Design and Manufacturing for Vehicle Body, College of Mechanical and Vehicle Engineering, Hunan University, Changsha 410082, China

^2^ School of Physics and Electronics, Hunan University, Changsha 410082, China

^3^ SZU-NUS Collaborative Innovation Center for Optoelectronic Science & Technology, International Collaborative Laboratory of 2D Materials for Optoelectronics Science and Technology of Ministry of Education, College of Optoelectronic Engineering, Shenzhen University, Shenzhen 518060, China

^4^ Department of Electrical and Computer Engineering, National University of Singapore, 4 Engineering Drive 3, Singapore 117583, Singapore

^5^ Engineering Product Development Pillar, Singapore University of Technology and Design, 8 Somapah Road, Singapore 487372, Singapore

^6^ Kirchhoff Institute for Physics, University of Heidelberg, Im Neuenheimer Feld 227, 69120 Heidelberg, Germany

^#^ These authors contribute equally to this work.

* Email: duanhg@hnu.edu.cn and [joel_yang@sutd.edu.sg](mailto:joel_yang@sutd.edu.sg).

In this work, we show the arrays of FP cavities with the pixel sizes of 500 nm. Here, we tapped into the thickness of 300 nm SiO_2_ overlay on these structures, Fig. S1a shows the palette after SiO_2_ film deposition. Compared to the sample without SiO_2_ coating, the colors of the palette are same to the original palette with SiO_2_ coating. Meanwhile, the filling density is defined as the proportion of the area occupied by the FP cavities. Fig. S1b illustrates the selection rule for the size of the filling density *D* in a 20-µm square while fixing the pixel size of 500 nm. Remarkably, a 20-μm square is divided into 1600 square pixels with a size of 500 nm and each square pixel is filled with a FP cavity with the size of *D*. We can obtain the value of *D* under various filling density associated with the equation (1).


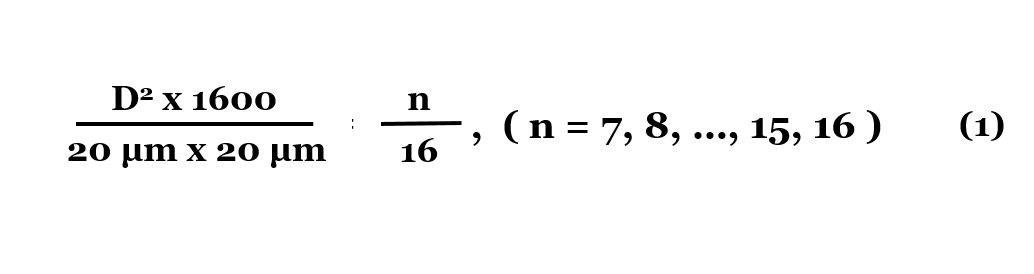

**Figure S1.** (a) Optical micrographs of the obtained color palette with SiO_2_ coating. Scale bars: 50 µm. (b) Schematic diagram of definition rule for the filling density *D* size with each pixel size of 500 nm.

Here, Optical micrographs of the color palette at different numerical apertures (NA) are shown in Fig. S2a to illustrate the angular dependence of proposed color tiles in this work. Using objective with different numerical apertures means that these tiles are exposed to different angle of incidence (θ). We can observe that the color of these tiles has no significant angle dependent variations with the increasing of incidence angle. For the TE polarization, the tilt angle increases from 12° up to 30°, the spectral responses for the polarizations have a slight difference as shown in Fig. S2b.

**Figure S2** (a) Optical micrographs of the obtained color palette under different numerical apertures. The specific numerical apertures are (i) 0.25 NA; (ii) 0.4 NA; (iii) 0.75 NA and (iv) 0.85 NA. Scale bars: 25 μm (i-iv). (b) Simulated transmission of the color filter for TE with various incidence angles (12° - 30°).

Figure S3 shows the simulated transfer characteristics without the top Ag layer. The positions of the dip are gradually red shifted when the filling density *D* gradually increases. This may be result from the wood anomaly or the diffraction effect of periodic nanostructures.

**Figure S3** Calculated transmission spectra of FP cavities (corresponding to the samples shown in Figure 3a) by adjusting the filling density of asymmetric FP cavities, but without the top Ag coating at normal incidence.

Figure S4a shows the simulated transfer characteristics with and without the quartz substrate. We found that the substrate is beneficial for improving the transmission slightly. We tapped into the thickness of 300 nm SiO_2_ overlay on these structures, which plays a crucial role in suppressing unwanted reflection. Figure S4b shows the experimental transmission spectra with and without 300 nm SiO_2_ coating, in which we investigated the effect of SiO_2_ coating on the transmittance of our designed structure the thickness of HSQ varies from 103 nm, 130 nm, 146 nm, 160 nm to 172 nm, and the *D* was fixed at 500 nm (indicated by a red box in Figure 2b). Obviously, the structure with SiO_2_ coating have a higher transmittance, the transmission of peaks is ~60% and the full width at half-maximum (FWHM) for the measured resonances is ∼37−41 nm.

**Figure S4** (a) simulation and (b) experimental transmission spectra of asymmetric FP cavities with varying *h* and the condition of *D* = 500 nm (the red highlighted column in Figure 2b), and the configuration with top SiO_2_ coating layer (dots) and without top SiO_2_ coating (solid lines) are shown.

By varying the exposure dose factor to tune the thickness of FP cavities during the greyscale lithography process, we can obtain vivid paintings with subtle saturation variations. Figure S5a shows the printing with the exposure dose varied from 42 μC/cm^2^ to 154 μC/cm^2^ at a step dose of 10 μC/cm^2^, Figure S5b shows the printing with the exposure dose varying from 48 μC/cm^2^ to 176 μC/cm^2^ and Figure S5c shows the printing with the exposure dose varied from 54 μC/cm^2^ to 198 μC/cm^2^.

**Figure S5** the pixel size of the printing is uniform *D* = 500 nm, (a) The exposure dose is varied from 42 μC/cm^2^ to 154 μC/cm^2^ at a step dose of 10 μC/cm^2^ and (b) the printing by varying from 48 μC/cm^2^ to 176 μC/cm^2^, and (c) the exposure dose is obtained from 54 μC/cm^2^ to 198 μC/cm^2^.
